# Innate and adaptive immune responses of snatch-farrowed porcine-colostrum-deprived pigs to *Mycoplasma hyopneumoniae* vaccination

**DOI:** 10.1186/s12917-014-0219-2

**Published:** 2014-09-20

**Authors:** Yanyun Huang, Andrea Ladinig, Carolyn Ashley, Deborah M Haines, John CS Harding

**Affiliations:** Department of Large Animal Clinical Sciences, Western College of Veterinary Medicine, University of Saskatchewan, 52 Campus Drive, Saskatoon, SK S7N 5B Canada; Department of Veterinary Microbiology, Western College of Veterinary Medicine, University of Saskatchewan, 52 Campus Drive, Saskatoon, SK S7N 5B Canada; Prairie Diagnostic Services Inc., Saskatoon, SK Canada

**Keywords:** Snatch-farrowed porcine-colostrum-deprived pigs, Animal model, *Mycoplasma hyopneumoniae*, Cytokine, Immunity, Fluorescent microsphere immunoassay

## Abstract

**Background:**

The snatch-farrowed porcine-colostrum-deprived (SF-pCD) pig model, in which neonates are raised on commercially available bovine colostrum, is an alternative model for porcine infectious disease research. It is not known if SF-pCD pigs possess growth performance and immunity comparable to conventional, farm-raised pigs. The current experiment compared growth performance and immune responses of SF-pCD pigs to their farm-raised siblings following *Mycoplasma hyopneumoniae* (*Mhyo*) vaccination. Twelve SF-pCD and 13 farm-raised siblings were vaccinated on day 7 (D7) and D26 of age. Body weights were measured once or twice weekly and average daily gain (ADG) was calculated. Peripheral blood mononuclear cells (PBMC) were isolated on D40. Cytokine secretion from PBMC stimulated with *Mhyo* antigen or phorbol myristate acetate plus ionomycin (PMA/Iono) was assessed using a multiplexed fluorescent microsphere immunoassay (FMIA). Additionally, interferon gamma (IFNγ) secretion from stimulated PBMC was assessed using ELISPOT. *Mhyo* IgG titers were measured by an ELISA in D40 sera.

**Results:**

Growth performance did not differ between groups before weaning, but SF-pCD pigs had higher ADG after weaning. In response to *Mhyo* stimulation, numbers of IFNγ secreting PBMC and levels of interleukin 8 (IL8) and IL10 in PBMC supernatants were significantly higher in SF-pCD pigs, as were *Mhyo* antibody levels in sera, and levels of IL1β, IL8 and IL12 in supernatants of PMA/Iono stimulated PBMC.

**Conclusions:**

Under the conditions of this experiment, SF-pCD pigs demonstrated superior growth performance and enhanced humoral and cell-mediated immunity following vaccination. Whether or not this reflects greater resistance or tolerance to infection is unknown but the ability to react positively to the vaccination provides evidence that SF-pCD pigs are a suitable alternative model for swine disease research.

**Electronic supplementary material:**

The online version of this article (doi:10.1186/s12917-014-0219-2) contains supplementary material, which is available to authorized users.

## Background

Reliable animal models are critical for reproducing infectious disease in experimental settings. Frequently used models for pigs include the conventional specific pathogen free (SPF) model, cesarean-derived colostrum-deprived (CDCD) model, and gnotobiotic model. Conventional SPF models are not always suitable for highly prevalent pathogens because the presence of maternal antibodies precludes challenge or vaccination of very young pigs. Both CDCD and gnotobiotic models require surgery to deliver piglets and sterile compartments in which they are raised. As gnotobiotic pigs are raised entirely in sterile compartments, the duration of experiments is limited because pigs may outgrow the allotted space. Further, gnotobiotic pigs fail to produce serum immunoglobulin (Ig)G and IgM antibodies to T-cell dependent and type-2 T-cell independent antigens, whereas pigs colonized by a single strain of *Escherichia coli* produce antibodies [1]. Accordingly, gnotobiotic pigs differ from conventional pigs immunologically. This raises questions regarding the applicability of gnotobiotic pig experiments to field situations. It was in this context that we optimized a previously published snatch-farrowed porcine colostrum-deprived (SF-pCD) pig model [2,3] to achieve 100% survival. This provided an alternative model for infectious disease research [4]. SF-pCD pigs were raised on a bovine-colostrum-based liquid diet before weaning, and a post-weaning diet that was free of porcine byproducts [4].

It is also unknown whether SF-pCD pigs are representative of conventional, farm-raised pigs in terms of their immunological responses. The objective of this research was to compare growth performance, and cellular and humoral immune responses following *Mycoplasma hyopneumoniae* (*Mhyo*) vaccination between SF-pCD pigs and their farm-raised siblings. Our results demonstrate that SF-pCD pigs have superior growth performance, humoral and cell mediated immunity following *Mhyo* vaccination compared to their siblings raised on a commercial farm.

## Methods

### Animal procedures

**Figure 1:**
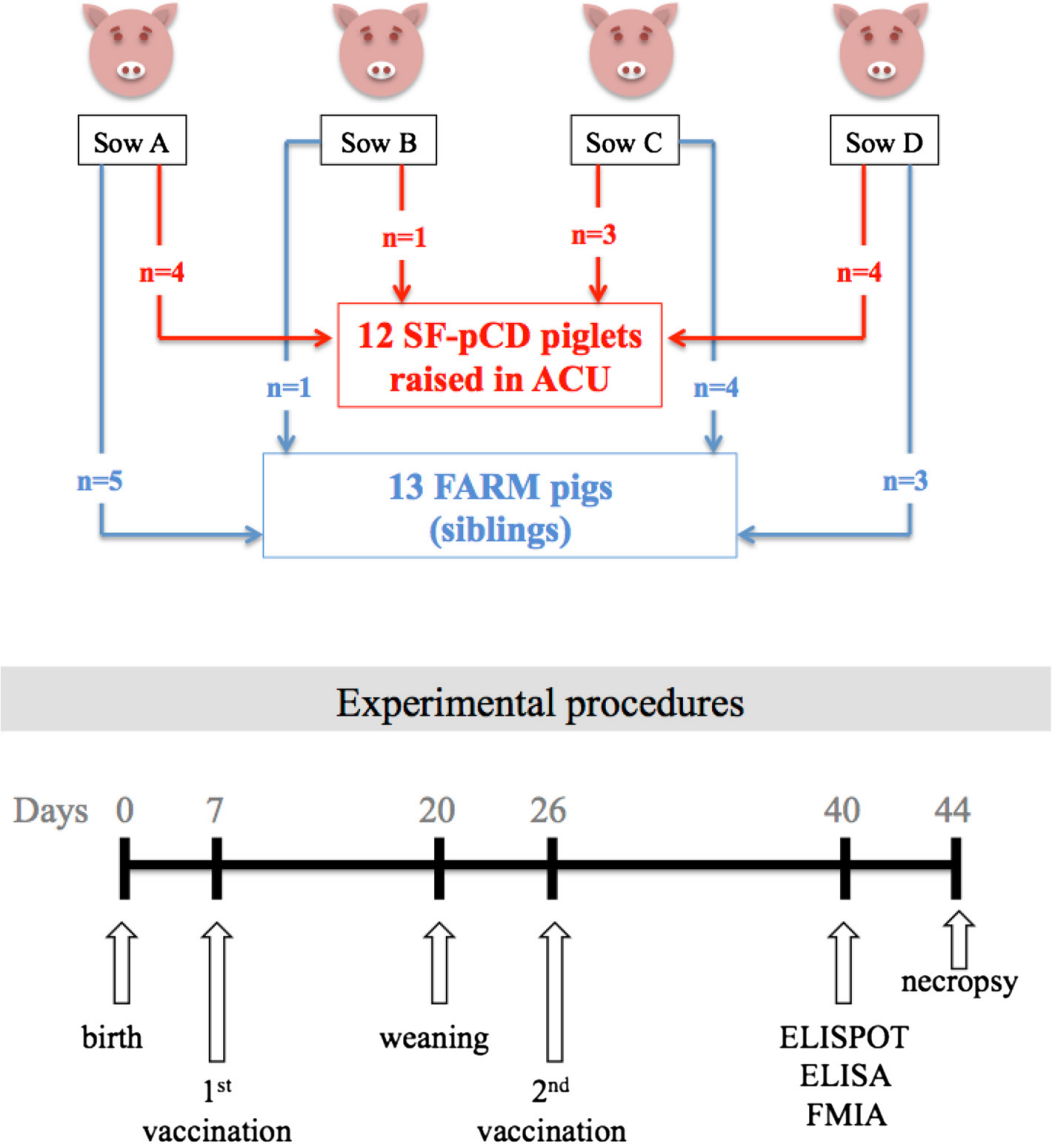
**Experimental design showing piglet selection and major events in the experiment.**

This work was approved by the University of Saskatchewan’s Animal Research Ethics Board and adhered to the Canadian Council on Animal Care guidelines for humane animal use (permit #20120031) and the REFLECT Guidelines for reporting of randomized control trials in livestock and food safety (Additional file 1). Twenty-five neonatal pigs were hygienically snatch-farrowed from four sows at the Prairie Swine Center Inc. (Saskatoon, Canada) as previously described [4]. The Prairie Swine Center is historically negative for *Mhyo* and sows were unvaccinated. Twelve SF-pCD pigs originating from PIC Camborough sows were raised using commercial bovine colostrum (HeadSTART and Calf’s Choice Total HiCal; The Saskatoon Colostrum Company Ltd., Saskatoon Canada) in a biosafety level 2 animal Care Unit as previously described [4]. Thirteen siblings (referred to hereafter as FARM), blocked by dam, sex and subjective birth sizes, remained on the farm to be raised by their biological sows. Non-experimental piglets were cross-fostered onto experimental dams as required to ensure litter sizes of 10–12 after farrowing. On day 2 (D2), 200 mg parenteral iron dextran (Ferroforte, Bimeda-MTC Animal Health Inc., Cambridge, ON) was administered. On D20, all pigs were abruptly transitioned to a dry starter diet free of porcine byproducts and remained on this diet until termination on D44. SF-pCD pigs were raised in groups of two in 1.23 m × 1.85 m pens with plastic slatted flooring and a two-hole liquid feeder. FARM pigs were weaned into two 2.5 m × 1.04 m nursery pens with slatted flooring in groups of six and seven. All pigs were weighed on D1 (day of birth was D0), twice weekly before weaning, and once weekly thereafter. On D7 and D26, pigs were vaccinated intramuscularly with 2 mL *Mhyo* bacterin (RespiSure, Zoetis Animal Health, Kirkland, QC). Blood samples were collected from the cranial vena cava on D1 and weekly thereafter (D7, 13, 20, 26, 33 and 40). The main events in this experiment are shown in Figure 1.

### Peripheral blood mononuclear cells (PBMC) isolation

Whole blood collected into sodium-heparin on D40 and diluted 1:1 with phosphate buffered saline (PBS; pH = 7.4) was overlaid on to Ficoll-Paque Plus (GE Healthcare Bio-Sciences Corp., Baie d’Urfe, QC) and separated by centrifugation at 400 *g* at 20°C for 30 min. After hypotonic lysis of red blood cells, PBMC were washed once with PBS and once with culture medium (Gibco® RPMI Media 1640, Life Technologies Inc., Burlington, ON) with 10% (v/v) fetal bovine serum (FBS) and 1% (w/v) penicillin-streptomycin (Sigma-Aldrich, Oakville, ON). Cells were stained with 0.4% Trypan blue (VWR International, Mississauga, ON) and counted on a hemocytometer.

### Interferon gamma (IFNγ) ELISPOT assay

MultiScreen filter plates (EMD Millipore Corp. Billerica, MA) were coated with 10 μg/mL mouse anti-porcine IFNγ monoclonal antibodies (Mabtech Inc. Cincinnati, OH), incubated overnight at 4°C, washed three times with PBS and blocked with culture medium at 37°C for 1 h. PBMC (8 × 10^5^) were dispensed into each well and incubated with 10 μg/mL whole cell sonicated *Mhyo* antigen (courtesy of M. Raymond, Iowa State University), 5 μg/mL Concanavalin A (ConA), or media alone for 40 h at 37°C in the presence of 5% CO_2_. Each sample was assayed in triplicate. Cells were removed and plates washed twice with double distilled water, thrice with PBS with 0.01% Tween 20 (PBST), then incubated with 1 μg/mL biotinylated mouse anti-bovine IFNγ mAb (Mabtech Inc.) for 1 h at room temperature. Plates were washed four times with PBST and incubated with 0.5 ng/mL streptavidin alkaline solution (Jackson ImmunoResearch Laboratories Inc., Westgrove, PA) in PBST with 1% bovine serum albumin (BSA) at room temperature for 45 min. Plates were washed three times with PBST and twice with PBS. Spots were developed by adding SIGMAFAST BCIP/NBT (Sigma-Aldrich Corp.) to each well according to the manufacturer’s instructions. The number of spots in each well was counted using an AID Elispot Reader ELRIFL07 (Autoimmun Diagnostika GMBH, Straßberg, Germany). Numbers of *Mhyo*-stimulated IFNγ secreting cells were calculated by subtracting the numbers of spots in media wells from those in *Mhyo* stimulated wells. Results were expressed as IFNγ secreting cells per million PBMC.

### Cytokine detection in supernatants of stimulated PBMC

PBMC (1 × 10^6^ cells) isolated on D40 were stimulated with 20 μg/mL *Mhyo* antigen or 10 ng/mL phorbol 12-myristate 13-acetate (PMA; Sigma–Aldrich) plus 250 ng/mL ionomycin (Sigma–Aldrich) (PMA/Iono). Supernatants harvested at 65 h post-stimulation were analyzed for interleukins (IL) 1β, IL4, IL8, IL10, IL12, and chemokine ligand 2 (CCL2) by multiplexed fluorescent microsphere immunoassay (FMIA) and IFNγ using an ELISA.

FMIA was performed as previously described [5] with several modifications. Briefly, individual capture antibodies were covalently coupled to magnetic beads (Bio-Rad Laboratories, Inc., Montreal, QC). The assay buffer was PBS, 1% BSA and 0.05% Na azide (PBS-BN). Cell culture medium, diluted in PBS-BN as appropriate, was used as a negative control. A standard curve was constructed using cytokine standards diluted appropriately in cell culture medium and PBS-BN. IL8 and CCL2 were tested in a duplex assay with the supernatants diluted 1:30 in PBS-BN. All other cytokines were tested in a four-plex assay with supernatants diluted 1:5 in PBS-BN. Samples and standards were incubated with magnetic beads in duplicate wells of a 96-well plate for 2 h. All incubations were performed in the dark at room temperature on a plate shaker rotating at 500 rpm. A 96-well plate washer (Bio-Plex Pro™ II Wash Station) was used to perform three washes with PBST after each incubation step. Secondary antibodies were added to each well before a 90 min incubation. Beads in each well were incubated in 50 μL of Strepavidin-R-Phycoerythrin (SAPE; Prozyme, Hayward, CA) for 30 min, washed and suspended in 100 μL PBST. Coupled microspheres were read in a Bio-Plex® 200 system (Bio-Rad Laboratories) and analyzed with Bio-Plex Manager software version 6.1 (Bio-Rad Laboratories). All mean fluorescence intensity (MFI) measurements were background corrected by subtracting the MFI of the negative control from the MFI for the respective analyte in each sample.

IFNγ concentrations in PBMC supernatants were tested using the Swine IFNγ Antibody Pair ELISA kit (Life Technology Inc.) according to the manufacturer’s instructions, except that the color substrate was KPL SureBlue Reserve TMB Microwell Substrate (Mandel Scientific Company Inc., Guelph, ON). Color was developed in the dark until the optical density of the highest concentration standard reached approximately 2.0 at a wavelength of 650 nm when read on a Vmax microplate reader (Molecular Devices, LLC., Downington, PA) without addition of stop solution. Supernatants were tested at a 1:5 dilution.

### Serum Mhyo antibody ELISA

Serum *Mhyo* antibody levels were determined using the IDEXX *Mhyo* ELISA (IDEXX Laboratories, Inc., Toronto, CA) at Biovet Inc. (Saint-Hyacinthe, QC). Additional titration was performed to establish end point titres for each sample. Briefly, a strong positive serum sample was serially diluted in PBS to determine the highest dilution that gave a positive ELISA result; in this case, 1:2560 that approximately corresponding to the ELISA positive cut off (0.40). The positive control sample was therefore assigned a titre of 2560. An eight dilution standard curve was then established (1:40 through 1:2560) and unknown samples diluted 1:40 were tested side-by-side with the standard curve on each plate. Finally, the S/P ratios were analyzed and converted to titres using curve-fitting software for ELISA analysis (MasterPlex® ReaderFit, Hitachi Solutions America Ltd., San Bruno, CA)

### Serum porcine and bovine IgG concentration

Serum bovine IgG concentrations were determined by radial immunodiffusion (RID) as previously described [4]. The porcine IgG RID assay was adapted from the bovine assay with had several modifications: the antibody was 3.0% goat anti-swine IgG (H + L) antibody (Jackson ImmunoResearch Laboratories Inc.) and the standard was purified swine IgG (Bethyl Laboratories Inc. Montgomery, TX). The half life of bovine IgG in SF-pCD pigs was calculated as described previously [4].

### Statistical analyses

Body weights (D1, D20 and D40), average daily gain (ADG) (overall, pre- and post-weaning), number of IFNγ secreting PBMC, cytokine concentrations in supernatants, serum *Mhyo* antibody levels, and serum porcine IgG concentration (all D40) were compared between groups using linear regression built by Generalized Estimation Equations (GEE) accounting for clustering of litters. All models used an identity link, Gaussian distribution and exchangeable correlation matrix with a robust variance estimator. Residuals were assessed for normality and homogeneity, and in cases where assumptions were violated, data was transformed (natural logarithm, logarithm base 10, or square root) and re-analyzed. If transformed data failed to satisfy the assumptions of linear regression, a non-parametric Mann-Whitney’s *U* test was used to assess group differences. All analyses were performed using IBM SPSS Statistics version 21. *P* < 0.05 was considered statistically significant, except for the cytokine analyses, where *P* < 0.01 was chosen in order to minimize the risk of type I error associated with testing multiple cytokines.

## Results

There were no significant group differences in body weight or ADG before weaning (Table 1). After weaning, SF-pCD pigs exhibited significantly increased ADG (*P* < 0.0001) and their final body weight was significantly greater than FARM pigs (*P* < 0.0001). As a result, the overall ADG of SF-pCD pigs was significantly greater than FARM pigs (*P* < 0.0001).

**Table 1:** **Body weights and average daily gains of SF-pCD and FARM pigs**

	**SF-pCD (n = 12)**		**FARM (n = 13)**		***P*** **value**
	**Mean**	**SD**	**Mean**	**SD**	
**Body weight (kg)**					
D1	1.45	0.28	1.39	0.19	ns
D20	6.58	0.70	6.71	0.88	ns
D40	17.14	1.57	12.45	1.77	<0.0001
**Average daily gain (kg/d)**					
Pre-weaning*	0.27	0.03	0.28	0.05	ns
Post-weaning*	0.53	0.05	0.29	0.08	<0.0001
Overall	0.40	0.04	0.28	0.04	<0.0001

*All pigs were transitioned to starter diet on 20 days of age.

On D40, the number of IFNγ secreting PBMC and *Mhyo* serum antibody levels were significantly greater in SF-pCD than FARM pigs (*P* < 0.0001 for IFNγ; *P* = 0.03 for *Mhyo;* Table 2). IL8 and IL10 secreted from *Mhyo* stimulated PBMC were also significantly greater in SF-pCD pigs (Table 3). PMA/Iono stimulated PBMC from SF-pCD pigs secreted significantly more IL1β, IL8 and IL12 than FARM pigs (Table 3). PMA/Iono failed to stimulate PBMC to secret CCL2 in this experiment (Table 3).

**Table 2:** **Day 40 mean** ***Mycoplasma hyopneumoniae*** **antibody titers, number of IFNγ secreting PBMC after** ***Mhyo*** stimulation, and porcine IgG concentration in SF-pCD and FARM pigs*

	**SF-pCD (n = 12)**		**FARM (n = 13)**		***P*** **value**
	**Mean**	**SD**	**Mean**	**SD**	
*Mhyo* IgG titers	447.9	208.3	336.8	290.5	0.03
IFNγ secreting cells/10^6^ PBMC	685	360	312	256	<0.0001
Porcine IgG (mg/mL)	2.9	1.2	4.6	0.8	0.05

Mhyo = *Mycoplasma hyopneumoniae*, IgG = immunoglobulin G.

*Blood was collected on D40, 33 days after the initial and 14 days after the booster vaccination.

**Table 3:** **Cytokine concentrations in supernatants of** ***Mycoplasma hyopneumoniae*** and PMA/Iono stimulated PBMC of SF-pCD and FARM pigs at 40 days of age*

	**SF-pCD (n = 12)**		**FARM (n = 13)**			
**Analytes**	**Mean**	**SE**	**Mean**	**SE**	**Statistical analyses**	***P*** **value** ^**§**^
***Mhyo*** **stimulated**						
IL1β	1510	399	1175	65	GEE	ns
IL4	2.0	0.7	4.6	1	GEE	0.042
IL8	15040	3327	8734	2197	GEE	**<0.0001**
IL10	188	54	119	35	GEE	**0.003**
IL12	2.6	1.7	3.3	2.5	GEE	ns
CCL2	282314	20251	223388	13847	GEE	0.081
IFNγ	58.0	18.8	93.1	48.5	GEE	ns
**PMA/Iono stimulated**						
IL1β	5346	806	1645	1084	GEE	**<0.0001**
IL4	58.0	21.5	42.2	2.7	GEE	ns
IL8	12156	2591	4347	4129	Mann–Whitney	**0.005**
IL10	22.6	1.0	45.9	11.4	GEE	0.032
IL12	72.0	7.0	2.2	3.5	GEE	**<0.0001**
CCL2	0	0	0	0	Mann–Whitney	1
IFNγ	3920	822	5032	1049	GEE	ns

Legend: *Mhyo* = *Mycoplasma hyopneumoniae,* PMA/Iono = phorbol 12-myristate 13-acetate plus 250 ng/mL ionomycin, GEE = Generalized Estimating Equations,

*Blood was collected 33 days after the initial and 14 days after the booster vaccination.

^§^ *P* < 0.01 considered statistically significant (bolded values).

**Figure 2:**
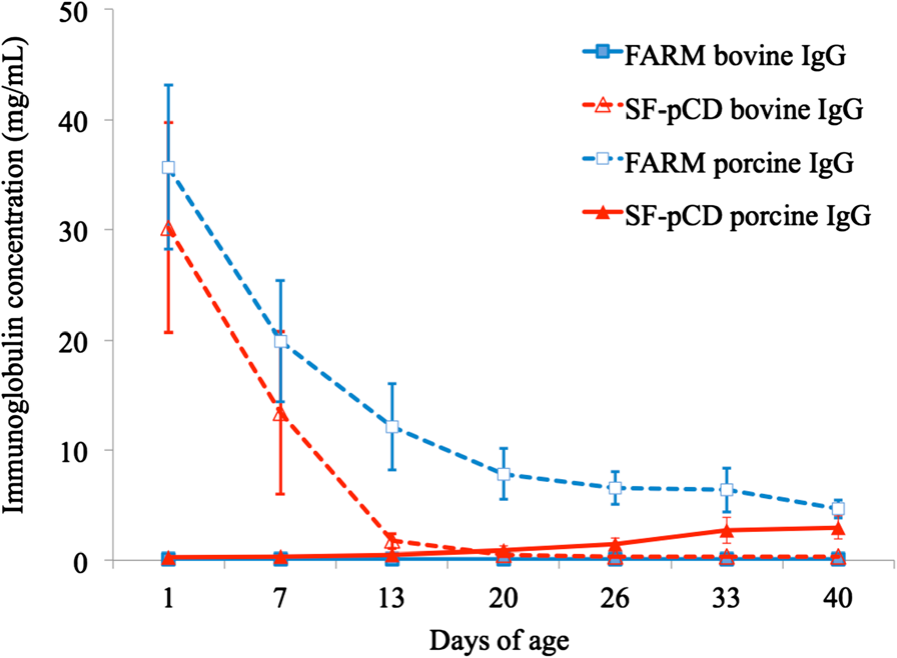
**Mean serum porcine and bovine IgG concentrations in SF-pCD (n = 12) and FARM pigs (n = 13).** The vertical bars represent standard deviations.

High concentrations of bovine IgG were present on D1 in SF-pCD pigs. This decayed rapidly over time with a calculated half-life of 6 d (Figure 2). Similarly, porcine IgG was present in abundance (35.7 ± 7.5 mg/L) in D1 sera of FARM pigs. An increase in porcine IgG in SF-pCD pigs was evident at D13 and gradually increased to approximately 3 mg/mL by D40 (Figure 2). Mean D40 porcine IgG concentration was significantly greater in FARM than SF-pCD pigs (Table 2).

## Discussion

This study yielded important results pertaining to the growth performance and immune responses of SF-pCD pigs compared to their conventionally raised siblings. The fact that pre-weaning growth rate of SF-pCD pigs did not differ from FARM pigs is noteworthy. SF-pCD pigs were bottle-fed for generally less than 48 h before learning to drink from a liquid feeder from which they were fed four times per day. This frequency of feeding was drastically lower than in conventionally reared pigs, which typically suckle more than 20 times per day [6]. Accordingly, it was a concern whether or not SF-pCD pigs would achieve growth rates similar to commercially raised pigs. Not only is this possible, but 100% survival should be expected using the established SF-pCD management protocol [4]. Being raised in a less competitive environment is one possible explanation why the ADG of SF-pCD pigs surpassed that of FARM pigs after weaning.

*Mhyo* was chosen as an exemplifier antigen because infection in susceptible pigs results in biologically relevant disease, commercial vaccines and antibody tests are available, and pigs free of *Mhyo* could be found locally. Since accurate titration of antibody levels is not possible with the IDEXX *Mhyo* ELISA, an additional titration step was performed to enable more accurate estimation of endpoint titres. Cell mediated responses were assessed in several ways including an IFNγ ELISPOT and by measuring IFNγ and IL12 secreted from PBMC following stimulation with *Mhyo* (recall) antigen and PMA/Iono. The latter is a potent mitogen that induces receptor-independent signal transduction involving conventional and novel protein kinase C (PKC) dependent pathways (mainly PKC isoforms α, β, ε, θ) [7-9]. Other cytokines reflecting innate immune responses were also measured in supernatants including: IL1β, a pro-inflammatory cytokine [10]; IL8, involved in neutrophil recruitment [11]; CCL2, involved in monocyte recruitment [11]; IL10, a regulatory cytokine [12]; and IL4, a traditional Th2 cytokine whose role is disputed in pigs [13].

There was no evidence to suggest that SF-pCD pigs were immunosuppressed. In fact, SF-pCD pigs had significantly more IFNγ secreting PBMC after *Mhyo* antigen stimulation and increased *Mhyo* antibody titers than FARM pigs, suggesting they had enhanced cellular and humoral responses. Although IFNγ levels in supernatants of *Mhyo* and PMA/Iono stimulated PBMC did not differ between groups, several other cytokines reflecting innate (IL1β, IL8), regulatory (IL10) and Th1 (IL12) responses were increased in SF-pCD. Although the reasons are not fully understood, group differences in the intestinal microbiota are a possible contributor. It is likely the intestinal microbial community structures of the two experimental groups differed as a result of differences in their respective environments and pre-weaning diets. Evidence in humans indicates that the neonatal intestinal microbial community regulates systemic immunity [14]. Moreover, pigs raised in environments with different levels of hygiene and intestinal microbial compositions exhibited different mucosal immunity characteristics [15]. The results of this experiment suggest that environmental factors may have affected the systemic immune response. Unfortunately, assessing differences in the intestinal microbiota was beyond the scope of this study.

Higher postweaning stress also may have contributed to group differences in postweaning immune responses. It is well known that pigs experience considerable stress shortly after weaning, demonstrated by a delayed weight gain and an increased serum cortisol concentration [16]. It is possible that SF-pCD pigs experienced less postweaning stress because pen density and feeding competition was lower than that experienced by FARM pigs. It should be noted, however, that increased cortisol levels in conventionally weaned farm pig return to normal within 6 d [16]. The timing of vaccinations in this experiment, on D7 and D26, were not in this postweaning stress period. Thus, if postweaning stress contributed to the findings in this study, the postweaning stress period may have extended beyond 6 d postweaning in the FARM pigs.

The composition of the milk and colostrum diets may have also contributed to the observed differences in the immune response of SF-pCD and FARM pigs. Colostrum is rich in immunoglobulin, but mature bovine and porcine milk contains much lower immunoglobulin concentration [17,18]. In addition to immunoglobulin, a large number of bioactive substances have been identified in bovine colostrum, such as insulin-like growth factors (IGF)-I and –II, epidermal growth factor (EGF), lactoferrin, and others [19]. By consuming bovine colostrum throughout the entire preweaning phase, SF-pCD pigs consumed more of these bioactive factors than FARM pigs. Although bovine and porcine colostrum contain similar levels of IGF-I, the mature milk of both species has about 10-fold less. Thus, SF-pCD pigs would have consumed more IGF-I throughout the suckling phase than FARM pigs that may have contributed to differences in the immune responses of SF-pCD relative to FARM pigs.

Finally, it is not known if the group differences in the magnitude of the immune response observed in this study are biologically relevant. Although SF-pCD pigs had significantly increased IFNγ response and *Mhyo* antibody titers, it was recently reported that increased serum *Mhyo* antibody titers but decreased IFNγ production by PBMC were associated with superior protection against *Mhyo* challenge [20]. This research has therefore generated several unanswered questions pertaining to variation in immune responses, which are worthy of pursuit.

## Conclusions

Under the conditions of this experiment SF-pCD pigs demonstrated superior growth performance and greater humoral and cell mediated immunity following *Mycoplasma hyopneumoniae* vaccination. Whether or not this would result in greater resistance to infection or less severe disease following exposure to *Mhyo* is unknown. SF-pCD pigs, however, are clearly not immunosuppressed and thus are suitable for porcine infectious disease research and could be used to address this and other questions relevant to the swine industry.

## Additional file

Additional file 1:
**REFLECT statement for present experiment.**
