# Supplementary material for: Innate and adaptive immune responses of snatch-farrowed porcine-colostrum-deprived pigs to Mycoplasma hyopneumoniae vaccination
Source: BMC Vet Res. 2014 Sep 20;10:219. doi: 10.1186/s12917-014-0219-2 (PMC4180582; doi:10.1186/s12917-014-0219-2)
Supplement: Additional file 1: — REFLECT statement for present experiment. [file 12917_2014_219_MOESM1_ESM.docx]

**Additional file 1**

**
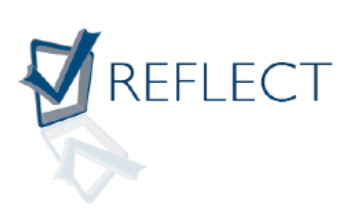
Checklist for REFLECT statement: Reporting guidelines for randomized control trials in livestock and food safety**. Bold text are modifications from the CONSORT statement description (Altman DG et al . Ann Intern Med 2001; 134(8):663-694).

| **Paper section and topic** | **Item** | **Descriptor of REFLECT statement item** | **Reported on Page #** |
| --- | --- | --- | --- |
| Title & Abstract | 1 | How **study units** were allocated to interventions ( eg, "random allocation," "randomized," or "randomly assigned"). **Clearly state whether the outcome was the result of natural exposure or was the result of a deliberate agent challenge.** | Allocation not reported in abstract (see Methods); method of exposure (vaccination) described in Abstract |
| Introduction Background | 2 | Scientific background and explanation of rationale. | Background section (pg 1-2) |
| Methods Participants | 3 | Eligibility criteria **for owner/managers and study units at each level of the organizational structure,** and the settings and locations where the data were collected. | Methods: Animal Procedures (pg 2) |
|  | 4 | Precise details of the interventions intended for each group, **the level at which the intervention was allocated,** and how and when interventions were actually administered. | Methods: Animal Procedures (pg 2-3) |
|  | 4b | Precise details of the agent and the challenge model, if a challenge study design was used. | Methods: Animal Procedures (pg 2) |
| Objectives | 5 | Specific objectives and hypotheses. **Clearly state primary and secondary objectives (if applicable).** | Background section (pg 2) |
| Outcomes | 6 | Clearly defined primary and secondary outcome measures and the levels at which they were measured, and, when applicable, any methods used to enhance the quality of measurements (eg, multiple observations, training of assessors). | Background section (pg 1-2), as well as appropriate sections in Methods (pg 2-4) |
| Sample size | 7 | How sample size was determined and, when applicable, explanation of any interim analyses and stopping rules. **Sample-size considerations should include sample-size determinations at each level of the organizational structure and the assumptions used to account for any non-independence among groups or individuals within a group.** | No sample size determinants included. Methods to deal with non-independence described in Methods: Statistical analyses (pg 4) |
| Randomization -- Sequence generation | 8 | Method used to generate the random allocation sequence **at the relevant level of the organizational structure**, including details of any restrictions (eg, blocking, stratification). | Subjects were blocked as described in Methods: Animal Procedures (pg 2) |
| Randomization -- Allocation concealment | 9 | Method used to implement the random allocation sequence **at the relevant level of the organizational structure**, (eg, numbered containers **~~or central telephone~~),** clarifying whether the sequence was concealed until interventions were assigned. | NA – see #8 above |
| Randomization -- Implementation | 10 | Who generated the allocation sequence, who enrolled **study units,** and who assigned **study units** to their groups **at the relevant level of the organizational structure**. | Methods: Animal Procedures (pg 2) |
| Blinding (masking) | 11 | Whether or not **~~participants~~** those administering the interventions, **caregivers** and those assessing the outcomes were blinded to group assignment. If done, how the success of blinding was evaluated**. Provide justification for not using blinding if it was not used.** | Impossible to blind animal care workers because groups were housed at different locations. All laboratory assays were blinded |
| Statistical methods | 12 | Statistical methods used to compare groups for all outcome(s); Clearly state the level of statistical analysis **and methods used to account for the organizational structure, where applicable;** methods for additional analyses, such as subgroup analyses and adjusted analyses. | Methods: Statistical analyses (pg 4) |
| Results  **Study** flow | 13 | Flow **of study units** through each stage **for each level of the organization structure of the study** (a diagram is strongly recommended). Specifically, for each group, report the numbers of **study units** randomly assigned, receiving intended treatment, completing the study protocol, and analyzed for the primary outcome. Describe protocol deviations from study as planned, together with reasons. | Methods: Animal Procedures (pg 2) and Figure 1 |
| Recruitment | 14 | Dates defining the periods of recruitment and follow-up. | NA |
| Baseline data | 15 | Baseline demographic and clinical characteristics of each group, **explicitly providing information for each relevant level of the organizational structure. Data should be reported in such a way that secondary analysis, such as risk assessment, is possible.** | Methods: Animal Procedures (pg 2) |
| Numbers analyzed | 16 | Number **of study units** (denominator) in each group included in each analysis and whether the analysis was by "intention-to-treat." State the results in absolute numbers when feasible (eg, 10/20, not 50%). | Tables 1, 2, 3 |
| Outcomes and estimation | 17 | For each primary and secondary outcome, a summary of results for each group, **accounting for each relevant level of the organizational structure**, and the estimated effect size and its precision (e.g., 95% confidence interval). | Tables 1, 2, 3 |
| Ancillary analyses | 18 | Address multiplicity by reporting any other analyses performed, including subgroup analyses and adjusted analyses, indicating those pre-specified and those exploratory. | NA |
| Adverse events | 19 | All important adverse events or side effects in each intervention group. | NA |
| Discussion Interpretation | 20 | Interpretation of the results, taking into account study hypotheses, sources of potential bias or imprecision, and the dangers associated with multiplicity of analyses and outcomes. **Where relevant, a discussion of herd immunity should be included. If applicable, a discussion of the relevance of the disease challenge should be included.** | Throughout Discussion |
| Generalizability | 21 | Generalizability (external validity) of the trial findings. | Discussion: last paragraph (pg 6) and Conclusions |
| Overall evidence | 22 | General interpretation of the results in the context of current evidence. | Conclusions |
